# Towards Precision and Balance in Selenium Nutrition: From Innovation to Application

**DOI:** 10.3390/nu18020320

**Published:** 2026-01-20

**Authors:** Jiaqiang Huang, Kongdi Zhu

**Affiliations:** Beijing Advanced Innovation Center for Food Nutrition and Human Health, Department of Nutrition and Health, China Agricultural University, Beijing 100083, China

Selenium (Se) is an essential trace element for human health, primarily functioning through its incorporation into selenoproteins, which play critical roles in antioxidant defense, immune regulation, and thyroid hormone metabolism [1,2]. While Se deficiency remains a global public health concern linked to various pathologies [3], the safe and effective dietary supplementation of Se continues to be a central focus in nutritional and food science. This Special Issue, “Se-Rich Products: Their Development and Regulation of Human Health,” compiles cutting-edge research that not only showcases innovative pathways for developing Se-enriched products but also delves into the complex mechanisms by which Se maintains health and counteracts disease. Collectively, these studies converge on a core theme: the dual imperatives of balance and precision are paramount to realizing the full health benefits of Se intervention.

The contributions in this issue deepen our understanding of the Se-health nexus from multiple dimensions. First, regarding biological mechanisms, several studies elucidate the role of Se in protection against specific environmental and metabolic stressors. Zhang et al. [4] systematically delineate the mechanisms through which Se and selenoproteins exert radioprotective effects via antioxidant actions, DNA repair, and immune enhancement, providing a theoretical foundation for developing radioprotective agents. Concurrently, the study by Li et al. [5] innovatively reveals the mediating role of oxidative stress (marked by albumin and vitamin D) in the association between dietary Se intake and cognitive function in middle-aged and older adults. This work directly links Se’s antioxidant properties to neurological outcomes, offering fresh insights for nutritional strategies aimed at mitigating cognitive decline. The theme of precision is further exemplified by studies on specific selenoproteins. An emerging study [6] reveals a novel mechanism by which the selenoprotein I regulates ferroptosis in colitis and colorectal cancer, independent of the well-known suppressor GPX4. Expanding this understanding of selenoprotein specificity, research on Selenoprotein V (SELENOV) has uncovered its critical in vivo functions. Studies demonstrate that SELENOV knockout in mice leads to increased fat accumulation, attenuated energy expenditure, and heightened susceptibility to pro-oxidant-induced liver injury and endoplasmic reticulum stress [7,8]. These findings establish SELENOV as a novel regulator of lipid metabolism, thermogenesis, and cellular redox resilience, highlighting the diverse and targeted roles individual selenoproteins play in metabolic health and stress response.

Complementing these mechanistic advances, the field is witnessing significant innovation in the development and validation of novel Se-rich products and sophisticated delivery systems. For instance, Zhu et al. [9] demonstrated the development of a functional product using the Se hyperaccumulator Cardamine violifolia, while concurrently highlighting the critical issue of co-accumulated heavy metals. This case underscores the imperative for rigorous safety assessments throughout the development of natural Se sources, a point reinforced by broader reviews advocating for full “soil-to-table” chain control to ensure safety and efficacy [10]. Beyond plants, the biotransformation capabilities of food-grade microorganisms offer a compelling strategy. Lactic acid bacteria (LAB) and bifidobacteria can accumulate inorganic Se and convert it into more bioavailable organic forms, such as selenocysteine and selenomethionine. Enriching these probiotics with Se not only creates a safe and nutritious source of this essential element but may also enhance the bacteria’s intrinsic functional properties, including antioxidative and anti-inflammatory activities. This approach represents a synergistic “dual-benefit” strategy for functional food design, targeting both improved Se nutrition and modulation of gut health [11]. In the realm of advanced delivery, Liu et al. presented an orally administrable, fungus-based Se microcarrier for protection against radiation-induced heart disease [12]. Advancing the frontiers of targeted therapy, Li et al. [13] developed a biomimetic platelet membrane-cloaked selenium nanoparticle (SeNP) system co-delivering a microRNA inhibitor for the precise treatment of hyperlipidemia. This system demonstrated prolonged circulation, liver-targeting capability, and dual-pathway efficacy, representing a sophisticated example of “precision nutrition” at the nanoscale.

The search for optimal Se forms and composites remains an active area of product innovation. Reviews on chitosan-based Se composites summarize how combining this biocompatible polymer with Se can enhance stability, gastrointestinal retention, and bioactivity, showcasing potential applications in functional foods and nutraceuticals [14]. Similarly, the field of selenopolysaccharides—organic compounds that combine Se with polysaccharide activities—is gaining attention. These compounds, derived from natural sources or through chemical synthesis and microbial enrichment, exhibit enhanced antioxidant, immunomodulatory, antitumor, and hypoglycemic activities compared to their separate components, offering a promising avenue for developing novel Se supplements [15].

This issue also places critical emphasis on the precision of Se status assessment and supplementation strategies. Accurate assessment of long-term Se status is fundamental. Emerging evidence suggests that biomarkers such as erythrocyte Se may provide a more reliable reflection of tissue reserves than short-term indicators like plasma Se. Equally vital is the recognition of Se’s narrow therapeutic window; both deficiency and excess intake—the latter potentially inducing adverse metabolic effects such as insulin resistance—underscore the imperative for dose-specific precision in intervention strategies [16].

The research frontier continues to expand into Se’s role against emerging health challenges. The protective role of Se in gastrointestinal health is further reinforced by studies utilizing advanced models. For example, research employing a gut-on-a-chip system revealed that biogenic SeNPs can ameliorate intestinal barrier oxidative damage by suppressing harmful epithelial-immune crosstalk, a finding validated in animal models [17]. Furthermore, the synthesis of highly stable, GroEL-stabilized biogenic SeNPs using probiotics has shown efficacy in alleviating experimental colitis, highlighting the potential of bio-engineered Se forms for managing inflammatory bowel diseases [18].

In summary, this Special Issue clearly illustrates that the science of Se nutrition is advancing in depth along the path of “mechanistic elucidation—product innovation—application evaluation.” Future research faces both challenges and opportunities. First, achieving “form-specific precision” requires a deeper understanding of the distinct pharmacokinetics, bioavailability, and bioactivity profiles of various Se species (e.g., inorganic selenite/selenate, organic selenomethionine/selenocysteine, and SeNPs) [19]. Second, the development of natural Se-rich foods and novel Se supplements must be accompanied by robust quality and safety control systems spanning from raw materials to final products [20]. The development of stable, bio-compatible forms such as the GroEL-stabilized Bio-SeNPs points the way forward. Third, leveraging more precise biomarkers like erythrocyte Se can facilitate the transition from population-based dietary recommendations to personalized nutrition guidance based on individual status. Fourth, active exploration of Se’s interventional strategies and specific applications in addressing defined health issues, such as radiation injury, environmental toxicant exposure, and metabolic syndrome, is warranted. Integrating advanced models like gut-on-a-chip into the research pipeline will further enhance the precision and human relevance of such explorations.

We sincerely thank all authors for their outstanding contributions to this Special Issue, as well as the reviewers for their expert evaluations and the editorial team for their steadfast support. We anticipate that this collection will serve not only as a compendium of current advances but also as a catalyst for future interdisciplinary research and innovation in “precision Se nutrition.” Ultimately, such endeavors are vital for translating scientific evidence into effective, safe, and personalized Se strategies to improve human health globally.
